# An annotated human blastocyst dataset to benchmark deep learning architectures for *in vitro* fertilization

**DOI:** 10.1038/s41597-023-02182-3

**Published:** 2023-05-11

**Authors:** Florian Kromp, Raphael Wagner, Basak Balaban, Véronique Cottin, Irene Cuevas-Saiz, Clara Schachner, Peter Fancsovits, Mohamed Fawzy, Lukas Fischer, Necati Findikli, Borut Kovačič, Dejan Ljiljak, Iris Martínez-Rodero, Lodovico Parmegiani, Omar Shebl, Xie Min, Thomas Ebner

**Affiliations:** 1 Software Competence Center Hagenberg, Data Science, Hagenberg, Austria; 2 American Hospital of Istanbul, In vitro fertilization lab, Istanbul, Turkey; 3 Viollier AG, Assisted Reproduction Technologies, Basel, Switzerland; 4 Hospital General Universitario de Valencia, In vitro fertilization lab, Valencia, Spain; 5 Semmelweis University, Department of Obstetrics and Gynecology, Division of Assisted Reproduction, Budapest, Hungary; 6 IbnSina and Banon IVF Centers, In vitro fertilization lab, Sohag, Egypt; 7 Bahceci Fulya IVF Centre Istanbul, In vitro fertilization lab, Istanbul, Turkey; 8 University Medical Centre Maribor, Department of Reproductive Medicine and Gynecological Endocrinology, Maribor, Slovenia; 9 Sestre Milosrdnice University Hospital Center, Department of Gynecology and Obstetrics, Zagreb, Croatia; 10 Universitat Autònoma de Barcelona, Laboratori de Fecundació In Vitro, Barcelona, Spain; 11 Next Fertility GynePro - NextClinic International, Bologna, Italy; 12 Kepler University Linz, Department of Gynecology, Obstetrics and Gynecological Endocrinology, Linz, Austria; 13 University Hospital Zurich, Department of Reproductive Endocrinology, Zurich, Switzerland

**Keywords:** Embryology, Computational science

## Abstract

Medical Assisted Reproduction proved its efficacy to treat the vast majority forms of infertility. One of the key procedures in this treatment is the selection and transfer of the embryo with the highest developmental potential. To assess this potential, clinical embryologists routinely work with static images (morphological assessment) or short video sequences (time-lapse annotation). Recently, Artificial Intelligence models were utilized to support the embryo selection procedure. Even though they have proven their great potential in different *in vitro* fertilization settings, there is still considerable room for improvement. To support the advancement of algorithms in this research field, we built a dataset consisting of static blastocyst images and additional annotations. As such, Gardner criteria annotations, depicting a morphological blastocyst rating scheme, and collected clinical parameters are provided. The presented dataset is intended to be used to train deep learning models on static morphological images to predict Gardner’s criteria and clinical outcomes such as live birth. A benchmark of human expert’s performance in annotating Gardner criteria is provided.

## Background & Summary

Medically Assisted Reproduction (MAR) started more than four decades ago and was primarily developed as a therapeutic treatment for couples suffering from tubal female factor infertility. Technologies as intracytoplasmic sperm injection (ICSI) were introduced and soon MAR was applicable to a variety of different infertility indications. So far, it is estimated that MAR techniques have resulted in the birth of over eight million children^1^. The tremendous impact of MAR in the field of medicine is illustrated by the fact that roughly 15 percent of the global population and therefore 48.5 million couples are affected by infertility^2,3^. Though huge efforts have been undertaken by research groups and *in vitro* fertilization (IVF) laboratories around the world, a live birth rate of less than 40 percent^1^ bears a huge potential for improvement^4^. In addition to the disappointment for couples in case of treatment failure, multiple cycles of extensive therapy, high expenses and physiological as well as psychological burden can cause stress and depression^5^. A regular treatment cycle in MAR is composed of several sequential steps, such as ovarian stimulation and puncture to collect cumulus-oocyte-complexes (COC), conventional IVF or ICSI for fertilization, short-time embryo culture *in vitro* up to 6 days to select the embryo of best prognosis for intrauterine transfer. Surplus embryos are vitrified for subsequent embryo transfers.

In the IVF laboratory the most crucial step is the selection of the embryo, preferably at blastocyst stage, with the highest implantation potential. Standardized schemes relying on both morphological and morphokinetic parameters of embryo development are commonly used to rate and rank the quality of the embryos and thus, the potential of the associated blastocysts to result in a successful pregnancy. This assessment is based either on observation of single static morphology images or time-lapse video sequences. The scoring of the developed blastocysts itself is performed by applying a standardized scheme such as the Gardner score, which rates the blastocyst expansion (EXP), as well as the quality of the inner cell mass (ICM) and trophectoderm (TE). This scheme^6^ outranged previous blastocyst scoring systems in a prospective study^7^ and its use was not only applied in multiple studies in order to increase IVF success rates, it is also recommended by an international expert group^8^. However, rating is prone to inter- and intra-observer variation. In addition, standard operating procedures (SOP) defined to implement the morphological assessment can vary between IVF centers and thus, introduce a bias.

Artificial Intelligence (AI) has gained attraction in IVF in order to generate a more standardized, unbiased approach to rate and select embryos for transfer^9–15^. Recent studies not only focused on selecting blastocysts for transfer, but also introduced trained models approaching an unbiased prediction of clinical parameters such as biochemical pregnancy, clinical pregnancy or life birth^11,12,16^. However, the comparison of results across studies imposes challenges as different scores for evaluation were used and study designs diverge^17^. To the best of our knowledge, no dataset is publicly available to be used for training and benchmarking AI-models with respect to Gardner scores or clinical parameters. In fact, there are only two publicly accessible datasets available: a dataset composed of bovine blastocysts^18^ and a dataset providing time-lapse movies of developing embryos including annotations of 16 different morphokinetic events^19^. Since bovine blastocysts differ from human blastocysts in morphological appearance, size and developmental speed^20^, this dataset^18^ is of little value for embryologists and researchers in the field of human MAR. The dataset containing videos of developing blastocysts is a valuable contribution towards deep learning-based assessment of blastocyst development phases, but cannot be used to predict scores according to the Gardner scheme or to infer a direct correlation to clinical parameters.

In this work, we want to bridge this gap by introducing a dataset consisting of images of human blastocysts including clinical annotations and expert-annotated Gardner criteria. Thereby, we are aiming to overcome the aforementioned pitfalls by providing i. a train- and test set split of all images including expert annotations of the Gardner criteria and the expert agreement (mean value and standard deviation), serving as reference to benchmark AI methods in comparison to human experts and ii. clinical parameters that can be used to support the development of AI architectures towards a prediction of these parameters from static blastocyst images. Thus, the dataset will support research groups to advance AI models towards an improvement of IVF success rates and towards a standardization of blastocyst selection for transfer to overcome intra- and inter-observer variability.

## Methods

### Participants and ethics

A total number of 2,344 blastocysts from 837 patients were included in this dataset. Blinding was employed during data collection. Informed consent was given and an ethical approval was obtained from the Ethics Committee of the Faculty of Medicine at the Johannes Kepler University in Linz, Austria (Nr. 1238/2021). All authors confirm that we have complied with all relevant ethical regulations.

### Embryo development

All oocytes collected during the study period were treated the same way. In detail, mature oocytes at metaphase II (MII) were either inseminated with conventional IVF or ICSI. Fertilization was controlled on the following day 1. *In vitro* culture of embryos through day 5 (blastocyst stage) was planned for all patients. For this purpose, a sequential culture medium was used (OS Cleav, OS Blast, Cooper Surgical, Denmark). The decision of which blastocyst to transfer was based on morphological appearance only. Transfer was done either on day 4 (if blastocysts were already available) or on day 5. Surplus morulae and blastocysts of good quality were vitrified (VitriStore, Gynemed, Germany). In approximately 8% of the cases a freeze-all strategy was sought due to a threatening ovarian hyperstimulation syndrome or suboptimal hormonal or uterine conditions and consequently these patients had exclusively vitrified-warmed blastocyst transfers.

### Blastocyst imaging

Over the 4-year study period photos of all blastocysts that were selected either for transfer or cryopreservation were taken. This was done using an Olympus IX50 (Vienna, Austria) microscope at a 400 times magnification while an imaging and archival software (Octax EyeWare, Vitrolife, Sweden) was used for documentation. Images were carefully taken to capture all three qualitative criteria of blastocyst stage (EXP, ICM and TE).

### Clinical annotations

Out of the 2,344 blastocysts used in this study, 752 (32.1%) were selected for fresh transfer (no vitrified-warmed cycles included). Clinical parameters associated with this subset are specified in the related data (number of COCs and MII oocytes, day of blastulation/embryo transfer, biochemical and clinical pregnancy as well as live birth).

### Gardner score consortium annotation

The Gardner scoring scheme is commonly used to rate blastocyst quality and potential as basis for the selection which blastocyst to select for transfer. To enable research groups to train AI models for predicting the Gardner criteria, an international consortium was formed and asked to annotate a subset of images, allowing to subsequently calculate a consensus agreement and to provide the Gardner scores for each image if defined.

#### Gardner criteria

The morphological grading system according to Gardner assigns a numerical score of 1–6 to blastocysts, further referred to as class of the score, based on their degree of expansion. Early blastocysts are blastocysts with beginning blastocoel formation (grade1) and blastocysts with a blastocele cavity ≤half of the size of the embryo (grade 2). It should be noted that at the early blastocyst stage (grades 1 and 2) ICM and TE are not yet clearly identifiable and thus, not defined. Full blastocysts (grade 3) are characterized by a blastocoel completely filling the embryo. Once the blastocyst starts to increase in size - a phase that is characterized by the thinning of the zona pellucida - grade 4 is reached (expanded blastocyst stage). Finally, the beginning of the hatching process or its completion is referred to as grades 5 and 6, respectively. Of note, no grade 6 blastocysts were seen in this study. From grade 3 onwards both cell lineages are prominent and can be distinguished and scored. Scoring of ICM and TE quality (grades A to C) is based on cell number and the degree of cohesion/compaction.

#### Consortium annotation

All 2,344 images were annotated with respect to the three Gardner criteria (EXP, ICM, TE) by a senior clinical embryologist with long-time experience, giving lectures and trainings on how to apply these criteria, who is further called Gardner-expert. In order to create a gold-standard test set, we selected a subset of 300 images, forming the test set. All images not assigned to the test set build the training set. Combined with the Gardner-expert annotations, this set is further called silver-standard training set. For test set assignment, images were randomly selected, but the selection algorithm was constrained such that images of all possible classes were included in a sufficient amount (*n* ≥ 7), for each of the three Gardner criteria. This constraint was necessary as the amount of images annotated with ICM and TE of class C were low in comparison to the overall dataset size (*n*_*ICM-C*_ = 23 and *n*_*TE-C*_ = 72, respectively). To create the gold-standard annotations without introducing an operator- or center-caused bias, an international consortium of 11 embryologists with at least six years of experience^21^ and working in 11 different clinics was formed and asked to annotate the test set images in addition to the Gardner expert. All consortium members have good knowledge of the Gardner scoring system but do not necessarily apply this scheme in their daily routine. To ensure a feasible workload for each of the embryologists, we created random splits of the 300 test set images such that i. each image was seen by at least five embryologists, and ii. each embryologist was assigned a total number of 150 images. For image annotation, the tool MakeSense (https://www.makesense.ai/) was used. MakeSense allows to view each image and to assign the respective class for each of the three Gardner criteria, using the predefined classes. In order to divide the task of image annotation into multiple sub-tasks that could be accomplished efficiently, each embryologist received five batches of the assigned 150 images (each batch consisted of 30 images) along with a manual on how to use the tool, and a list containing a definition of possible classes for each of the three criteria (EXP: Class 1 to 5; ICM and TE: Class A, B, C, Not defined). The embryologists were then asked to score each image in a batch, for all batches, using the class “Not defined” for ICM and TE criteria in case of EXP 1 or 2. If a class value could not be determined (e.g. because one image detail was not clearly visible), the experts were asked to not assign a class (empty value). Upon annotation, MakeSense allows to export a comma separated values (CSV) file including the class labels assigned to the images, for each batch. We collected all CSV files and merged them to obtain the annotations for all embryologists.

#### Refined consortium agreement

**Fig. 1:**
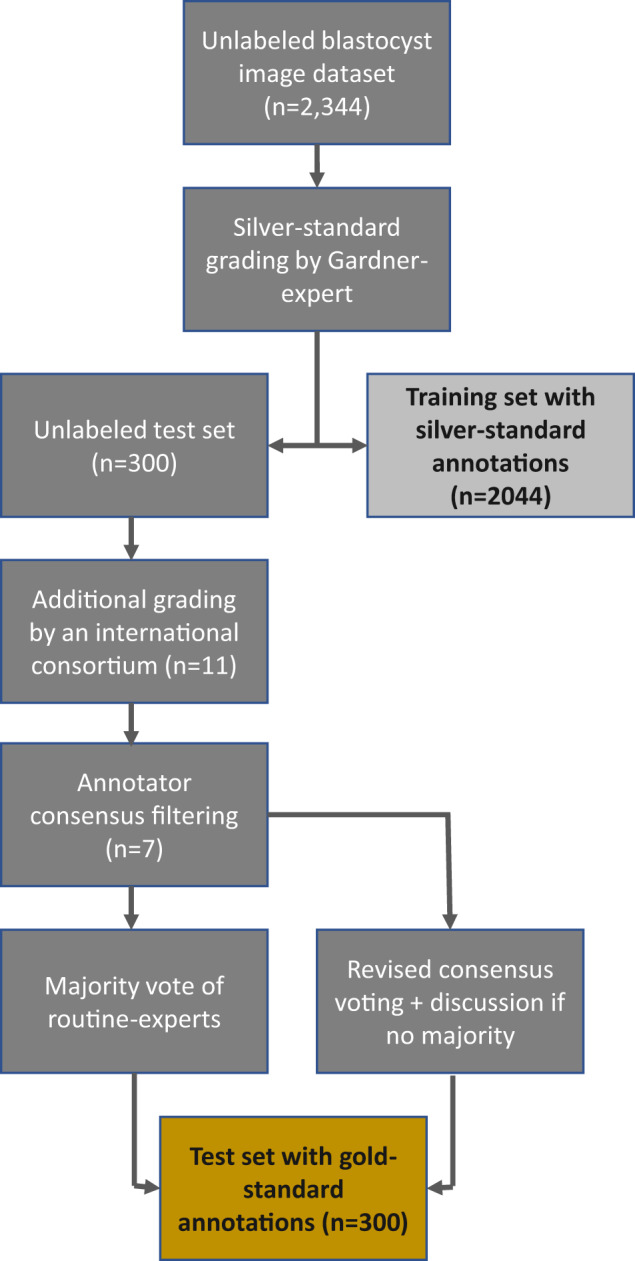
Workflow of creating the silver-standard training set and the gold-standard test set involving the Gardner-expert and an international consortium consisting of experienced clinical embryologists.

As previously stated, the consortium consisted of experienced clinical embryologists, although not all of them use and apply the Gardner criteria in their daily routine because they rather rely on modified Gardner scoring or in-house grading systems. To refine the consortium, we compared the accuracy of each embryologist’s annotations to the Gardner-expert annotations, for each of the three criteria. If an image was not rated for one of the criteria, this image was not considered while calculating the accuracy scores. We then excluded all embryologists whose accuracy for at least one of the three Gardner criteria was below 0.5 when compared to the Gardner-expert annotations (*n* = 5). From the remaining group including the Gardner-expert (*n* = 7), further called routine-experts, we formed the majority vote for each image and for each of the three criteria. In case no majority vote could be formed for one or more of the Gardner criteria, the image and the criteria were noted to be re-annotated. In total, 89 images had to be re-annotated. To do so, a subgroup of 6 of the embryologists remaining after consensus filtering voted for the respective Gardner criteria in two separate session (the tool Ahaslides was used to interactively collect polls for the class values for the missing criteria, https://ahaslides.com/), for each of these images. In case no majority was achieved, the class values were discussed until all embryologists could agree to a consensus. The final workflow applied to create the silver-standard training set and the gold-standard test set is illustrated in Fig. 1.

## Data Records

The dataset assigned with this paper is hosted at the Figshare repository^22^. The dataset contains 2,344 blastocyst images, provided in Portable Network Graphics (PNG) format. In addition, three CSV-files are provided, containing i. the assignment of images to the Gardner criteria training set including silver-standard annotations of all three criteria (EXP, ICM, TE), ii. the assignment of images to the Gardner criteria test set including gold-standard annotations of all three criteria and iii. the assignment of images to the clinical dataset and thus, the images of blastocysts that had been transferred including their clinical annotations. Class coding of all annotations are described in Table 1.

**Table 1:** Parameters provided in the annotations files. EXP = Expansion, ICM = Inner cell mass, TE = Trophectoderm, revised_cons = Revised consensus vote.

Criteria	Description	Possible values	annotation-file(s)
Image	Name of assigned image	text	all files
EXP silver	Expansion (silver-standard)	0 = 1, 1 = 2, 2 = 3, 3 = 4, 4 = 5	GTS, CA
ICM silver	Inner cell mass (silver-standard)	0 = A, 1 = B, 2 = C, 3 = not defined	
TE silver	Trophectoderm quality (silver-standard)	0 = A, 1 = B, 2 = C, 3 = not defined	
EXP gold	Expansion (gold-standard)	0 = 1, 1 = 2, 2 = 3, 3 = 4, 4 = 5,	GTG
		NA = not assessable	
ICM gold	Inner cell mass (gold-standard)	0 = A, 1 = B, 2 = C, ND = not defined,	
		NA = not assessable	
TE gold	Trophectoderm quality (gold-standard)	0 = A, 1 = B, 2 = C, ND = not defined	
		NA = not assessable	
EXP agreement	Grade of routine-expert agreement (EXP)	0..1, revised_cons	
ICM agreement	Grade of routine-expert agreement (ICM)	0..1, revised_cons	
TE agreement	Grade of routine-expert agreement (TE)	0..1, revised_cons	
EXP agreement desc	Ratio of routine-expert agreement (EXP)	#*agreeing expert*s/#*all experts*,	
		revised_cons	
ICM agreement desc	Ratio of routine-expert agreement (ICM)	#*agreeing experts*/#*all experts*,	
		revised_cons	
TE agreement desc	Ratio of routine-expert agreement (TE)	#*agreeing experts*/#*all experts*,	
		revised_cons	
d	Day	4, 5	CA
AMH	Anti-Mullerian Hormone (ng/ml)	0.08–19.40	
Age	Female age (years)	20–45	
Endo	Height of endometrium at ovulation induction (mm)	4–20	
COC	Number od cumulus-oocyte-complexes	2–26	
MII	Number of mature metaphase II oocytes	1–22	
SS	Biochemical pregnancy (positive hCG)	0..1	
HA	Ongoing pregnancies with clinical heart activity	0..1	
LB	Live birth	0..1	

GTS = Gardner train silver.csv, GTG = Gardner test gold.xlsx, CA = Clinical annotations.csv.

### Deep learning model training

To provide a deep learning baseline for future methods to compare with, we trained three recent deep learning architectures for the task of blastocyst image grading: an XCeption architecture^23^ as this architecture is commonly used in related publications on automated blastocyst grading^11,24^, and two vision transformer architectures (Deit transformer^25^, Swin transformer^26^), as vision transformers have been proven to achieve results comparable to conventional convolutional neural network architectures in image classification tasks while requiring substantially fewer resources^27,28^. We implemented stochastic weight averaging gaussian (SWA-G), a method used to reflect and calibrate uncertainty representation in Bayesian deep learning^29^. It is based on modelling a Gaussian distribution for each networks’ weight and applying it as a posterior over all neural network weights to perform Bayesian model averaging. All architectures were then trained on the silver-standard dataset using equal hyper-parameters: input size 224*x*224*x*3, SWA-G starting after 30 epochs, learning rate for SWA 2.5e-04, number of epochs 60, batch size 64, adam optimizer, cosine learning rate scheduler, warmup learning rate 1e-06, learning rate 5e-04, warmup epochs 5, imagenet standard color normalization, data augmentation: random crop and scale, horizontal flip, vertical flip, rotation. All hyper-parameters were obtained experimentally on a reduced dataset and fixed for all training runs. For each of the architectures, we trained separate models for each of the three Gardner criteria (expansion, inner cell mass quality, trophectoderm quality).

## Technical Validation

**Fig. 2:**
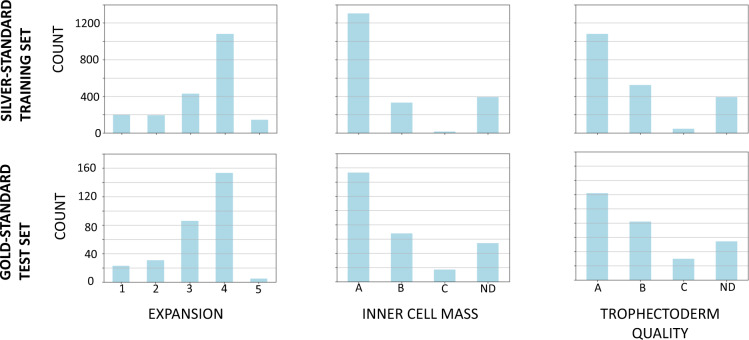
Class assignment distribution of the Gardner criteria within the silver-standard training set and the gold-standard test set. Not assessable class values not reported.

The selection of blastocyst images was performed by a team including the Gardner-expert. Only images fulfilling the quality criteria sharpness and homogeneous illumination were chosen. The subset of images forming the Gardner criteria test set were carefully selected such that images of each possible class were included, for all the three criteria. The minimum number of images per class included in the test set after annotator consensus filtering is seven. The assignment of images to all classes in the training- and the test set are depicted in Fig. 2. Upon annotation by an international consortium consisting of experienced clinical embryologists, we refined the consortium and calculated the majority vote as described in Section *Refined consortium agreement*.

### Inter-annotator agreement

**Fig. 3:**
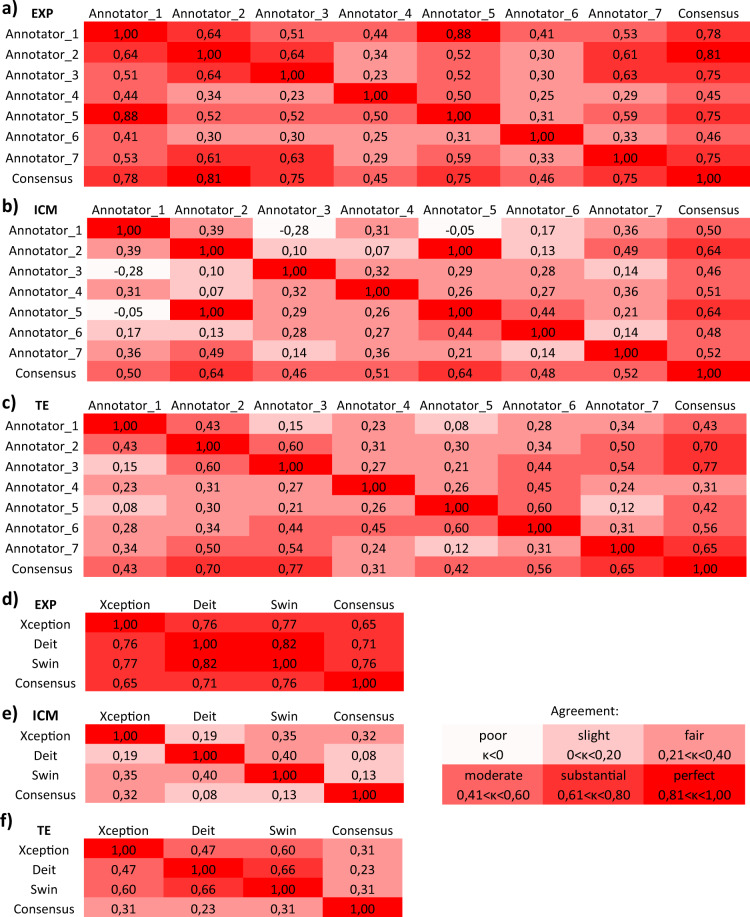
Inter-Annotator agreement (**a**–**c**) and deep learning baseline agreement (**d**–**f**) with the consensus calculated using Cohen’s Kappa-score.

We then calculated the inter-annotator agreement using Cohen’s Kappa score^30^, see Fig. 3. The Kappa score reflects the inter-annotator reliability in contrast to agreement occuring by chance. The resulting scores are set in a range between −1 and 1, where 1 is a perfect agreement between annotations and values below 0 are considered as poor agreement. According to Landis and Koch^31^ we use the following classification to rate the resulting Kappa scores *κ*: *κ* < 0: poor agreement, 0 < *κ* < 0.20: slight agreement, 0.21 < *κ* < 0.40: fair agreement, 0.41 < *κ* < 0.60: moderate agreement, 0.61 < *κ* < 0.80: substantial agreement and 0.81 < *κ* < 1: perfect agreement. As can be observed in Fig. 3, the agreement between expert embryologists w.r.t. expansion is fair to perfect. For trophectoderm quality, the agreement is slight to substantial, whereas for inner cell mass quality, the agreement is slight to substantial, with an exception between Annotator 1 compared to Annotators 3 and 5 with a poor agreement. When observing the agreement of annotators to the consensus vote, the agreement is fair to perfect, for each of the three criteria.

### Benchmark for deep learning models

We next calculated metrics from expert embryologist annotations to serve for benchmarking deep learning models. To provide a model baseline, we trained three state-of-the-art deep learning architectures on the silver-standard training set as described in section *Deep learning model training*. We report the accuracy and the mean and standard deviation of the class-weighted average of precision, recall and F1-score, including the scores achieved by the trained deep learning models, see Table 2. Recall, precision and the resulting F1 score are consistently between 0.70 and 0.83 for the expert embryologists. The deep learning baseline (XCeption, Deit- and Swin transformer) surpass these scores for the criteria expansion (0.77 to 0.85), but achieve lower scores for the inner cell mass and the trophectoderm (0.51 to 0.69). These results further serve as benchmark for AI models in predicting the Gardner criteria by comparison to the baseline results and the expert embryologists.

**Table 2:** Mean value and standard deviation of the accuracy and the weighted class-average of precision, recall and F1-score of the expert embryologists remaining after consensus filtering, compared to the consensus vote.

Gardner criteria	Subject	accuracy	avg-prec	avg-rec	avg-f1
Expansion	mean ± std experts	0.78 ± 0.12	0.83 ± 0.07	0.78 ± 0.12	0.79 ± 0.10
	XCeption	0.78	0.77	0.78	0.77
	Deit transformer	0.82	0.81	0.82	0.81
	Swin transformer	0.85	0.85	0.85	0.84
Inner cell mass	mean ± std experts	0.74 ± 0.06	0.76 ± 0.04	0.74 ± 0.06	0.74 ± 0.05
	XCeption	0.69	0.63	0.69	0.65
	Deit transformer	0.63	0.54	0.63	0.52
	Swin transformer	0.65	0.54	0.65	0.56
Trophectoderm	mean ± std experts	0.70 ± 0.14	0.78 ± 0.06	0.70 ± 0.14	0.71 ± 0.12
	XCeption	0.62	0.56	0.62	0.56
	Deit transformer	0.58	0.59	0.58	0.51
	Swin transformer	0.62	0.62	0.62	0.55

The results of the deep learning models is included as baseline for algorithmic benchmark.

## Data Availability

All scripts used to create the data splits and the final results are provided in a GitHub repository (https://github.com/software-competence-center-hagenberg/Blastocyst-Dataset).

## References

[1] Calhaz-Jorge C (2020). Survey on ART and IUI: legislation, regulation, funding and registries in European countries. Human Reproduction Open.

[2] Sharlip ID (2002). Best practice policies for male infertility. Fertility and Sterility.

[3] Mascarenhas MN, Flaxman SR, Boerma T, Vanderpoel S, Stevens GA (2012). National, Regional, and Global Trends in Infertility Prevalence Since 1990: A Systematic Analysis of 277 Health Surveys. PLoS Medicine.

[4] Centers for Disease Control and Prevention. Assisted Reproductive Technology Fertility Clinic Success Rates Report - 2017. **17**, 105–116 (2017).

[5] Chachamovich JL (2010). Psychological distress as predictor of quality of life in men experiencing infertility: A cross-sectional survey. Reproductive Health.

[6] Gardner DK, Lane M, Stevens J, Schlenker T, Schoolcraft WB (2000). Blastocyst score affects implantation and pregnancy outcome: Towards a single blastocyst transfer. Fertility and Sterility.

[7] Balaban B, Yakin K, Urman B (2006). Randomized comparison of two different blastocyst grading systems. Fertility and Sterility.

[8] Balaban B (2011). The Istanbul consensus workshop on embryo assessment: Proceedings of an expert meeting. Human Reproduction.

[9] Enatsu N (2022). A novel system based on artificial intelligence for predicting blastocyst viability and visualizing the explanation. Reproductive Medicine and Biology.

[10] Loewke, K. *et al*. Characterization of an artificial intelligence model for ranking static images of blastocyst stage embryos. *Fertility and Sterility* 1–7, 10.1016/j.fertnstert.2021.11.022 (2022). 10.1016/j.fertnstert.2021.11.022 34998577

[11] Bormann CL (2020). Performance of a deep learning based neural network in the selection of human blastocysts for implantation. eLife.

[12] Tran D, Cooke S, Illingworth PJ, Gardner DK (2019). Deep learning as a predictive tool for fetal heart pregnancy following time-lapse incubation and blastocyst transfer. Human Reproduction.

[13] Kragh, M. F., Rimestad, J., Berntsen, J. & Karstoft, H. Automatic grading of human blastocysts from time-lapse imaging. *Computers in Biology and Medicine* **115**, 10.1016/j.compbiomed.2019.103494 (2019). 10.1016/j.compbiomed.2019.103494 31630027

[14] Thirumalaraju, P. *et al*. Evaluation of deep convolutional neural networks in classifying human embryo images based on their morphological quality. *Heliyon* **7**, 10.1016/j.heliyon.2021.e06298 (2021). 10.1016/j.heliyon.2021.e06298 PMC7907476 33665450

[15] Wang S, Zhou C, Zhang D, Chen L, Sun H (2021). A deep learning framework design for automatic blastocyst evaluation with multifocal images. IEEE Access.

[16] Goyal, A., Kuchana, M. & Ayyagari, K. P. R. Machine learning predicts live-birth occurrence before *in-vitro* fertilization treatment. *Scientific Reports* **10**, 10.1038/s41598-020-76928-z (2020). 10.1038/s41598-020-76928-z PMC7708502 33262383

[17] Sfakianoudis K (2022). Reporting on the Value of Artificial Intelligence in Predicting the Optimal Embryo for Transfer: A Systematic Review including Data Synthesis. Biomedicines.

[18] Rocha, J. C. *et al*. Data Descriptor: Automatized image processing of bovine blastocysts produced *in vitro* for quantitative variable determination. *Scientific Data* **4**, 10.1038/sdata.2017.192 (2017). 10.1038/sdata.2017.192 PMC5735923 29257125

[19] Gomez T (2022). A time-lapse embryo dataset for morphokinetic parameter prediction. Data in Brief.

[20] Bó GA, Mapletoft RJ (2013). Evaluation and classification of bovine embryos. Anim. Reprod..

[21] Kovačič B (2020). ESHRE Clinical Embryologist certification: the first 10 years†. Human Reproduction Open.

[22] Kromp F (2022). figshare.

[23] Chollet, F. Xception: Deep learning with depthwise separable convolutions. In *Proceedings of the IEEE conference on computer vision and pattern recognition*, 1251–1258, 10.4271/2014-01-0975 (XCeption, 2017).

[24] Zaninovic N, Rosenwaks Z (2020). Artificial intelligence in human in vitro fertilization and embryology.

[25] Touvron, H. *et al*. Training data-efficient image transformers distillation through attention. *Proceedings of the 38th International Conference on Machine Learning, PMLR* (2020).

[26] Liu, Z. *et al*. Swin Transformer. *2021 IEEE/CVF International Conference on Computer Vision (ICCV)* 9992–10002 (2021).

[27] Khan S (2022). Transformers in Vision: A Survey. ACM Computing Surveys.

[28] Dosovitskiy, A. *et al*. An Image is Worth 16 × 16 Words: Transformers for Image Recognition at Scale. *arXiv preprint* (2020).

[29] Maddox WJ, Garipov T, Izmailov, Vetrov D, Wilson AG (2019). A simple baseline for Bayesian uncertainty in deep learning. Advances in Neural Information Processing Systems.

[30] Cohen J (1960). A coefficient of agreement for nominal scale. Educ. Psychol. Meas..

[31] Landis JR, Koch GG (1977). The Measurement of Observer Agreement for Categorical Data. Biometrics.

